# Clinical outcomes of xeno-free expansion and transplantation of autologous ocular surface epithelial stem cells via contact lens delivery: a prospective case series

**DOI:** 10.1186/s13287-015-0009-1

**Published:** 2015-03-12

**Authors:** Samantha Bobba, Sharron Chow, Stephanie Watson, Nick Di Girolamo

**Affiliations:** School of Medical Sciences, University of New South Wales, High Street, Kensington, Sydney, 2052 Australia; Save Sight Institute, University of Sydney, 8 Macquarie Street, Sydney, 2000 Australia; Sydney Eye Hospital, 8 Macquarie Street, Sydney, 2000 Australia; Faculty of Medicine, University of New South Wales, High Street, Kensington, Sydney, 2052 Australia

## Abstract

**Introduction:**

Depletion of limbal stem cells leads to a debilitating condition known as limbal stem cell deficiency, characterised by impaired corneal wound healing and poor vision. The aim of this study was to determine whether delivering progenitor cells on a contact lens is a viable and effective alternative to current transplantation techniques, which are complicated by biological and xenogeneic materials.

**Methods:**

Sixteen eyes of 16 patients who had total (n = 14) and partial (n = 2) limbal stem cell deficiency (chemical burns, five eyes; iatrogenic causes, four eyes; aniridia, three eyes; trachoma-induced, two eyes; contact lens over-wear, one eye; and cicatrising conjunctivitis, one eye) and who had failed prior therapy were recruited prospectively into the study. Autologous limbal (n = 7) or conjunctival epithelial (n = 9) biopsies were harvested from patients and placed on the concave surface of silicone hydrogel contact lenses. Cells were expanded in culture with autologous serum and transplanted onto the ocular surface.

**Results:**

Restoration of a transparent avascular and clinically stable corneal epithelium was attained in 10 of 16 eyes (63%) at a median follow-up time of 2.5 years (range of 0.8 to 5.8 years). Although minor complications occurred in two eyes of two patients because of contact lens insertion or removal, these were not associated with long-term sequelae.

**Conclusions:**

This is the first and largest study to evaluate the mid-term outcomes of autologous limbal/conjunctival stem cell transplantation via a US Food and Drug Administration-approved contact lens, demonstrating that delivery of ocular progenitor cells via this procedure offers a viable, effective, and xeno-free alternative to current transplantation methodologies.

**Trial registration:**

Australian New Zealand Clinical Trials Registry ACTRN012607000211460. Registered 17 April 2007.

## Introduction

The corneal epithelium is maintained by stem cells (SCs) presumed to reside in the transitional zone between the cornea and conjunctiva, also known as the limbus [1]. Depletion of limbal epithelial stem cells (LESCs) through damage to their microenvironment (the niche) or aberrant functional modification can result in limbal stem cell deficiency (LSCD), a disease characterised by impaired corneal wound healing, loss of vision, and chronic pain [2]. LSCD is classified as either partial (involving a sector) or total (affecting the entire cornea) and encompasses a range of aetiologies. Surgical treatment via keratoplasty is deemed ineffective as these patients lack the SCs needed to re-epithelialise their corneal surface.

Since the pioneering work of Kenyon and Tseng [3] (1989) on limbal tissue transplantation, the field has grown exponentially. Moreover, substantial research efforts have been devoted to developing carrier substrates that facilitate cell expansion *ex vivo* and integration during transplantation. To date, the most commonly used substrate for LESC transplantation is human amniotic membrane (HAM), and reported success rates in patients with LSCD range from 46% to 100% [4-6]. Although HAM is non-immunogenic, anti-angiogenic and contains SC support factors, its disadvantages include its semi-opaqueness, donor-to-donor variability, expensive screening, and strict guidelines for preparation and storage [7-9]. Fibrin has been trialed as an alternative carrier; however, its short setting time poses difficulties in manipulating the membrane film during surgery [10,11]. Several other biomaterials have been trialed in *ex vivo* or animal models, or both, albeit to differing degrees. These scaffolds include silk fibroin [12,13], collagen shields [14], anterior human lens capsule [15], and silicone hydrogen contact lenses (CLs) [16-18]. Recent studies have shown that cells labeled *ex vivo* can adhere to CLs and are able to successfully transfer, survive, and proliferate after delivery [19,20]. Moreover, our clinical trial using this system showed that CLs could be used as a carrier and substrate for *in vivo* delivery of ocular surface cells, with a 100% success rate recorded in three patients with LSCD 12 months after the procedure [21]. Since we first reported our technique, biodegradable membranes which eliminate the need to remove the carrier have also been developed; however, they are still in the preliminary stages of trialing [22]. Advantages of CLs as a substrate include its transparency, mechanical stability, cost-effectiveness, and non-immunogenic nature. Currently, comparisons between different transplantation methodologies for managing LSCD are lacking and there is an increasing need for xeno-free expansion to meet the increasingly strict regulatory demands. Herein, we report the short- to mid-term outcomes of using CLs for xeno-free culture and expansion and as a carrier for ocular surface SC transplantation in 16 patients with LSCD.

## Methods

### Clinical trial

The clinical trial was registered in Australia (ACTRN-012607000211460) and approved by the South Eastern Sydney Local Health District Human Research Ethics Committee (SESLHD HREC-07/025). The SESLHD Executive Committee approved the follow-up arm of the trial (HREC-13/139). All components of this study were carried out in accordance with the Declaration of Helsinki. The protocol for using human cells and tissue was approved by the University of New South Wales Human Research Ethics Committee (HREC-06290). Informed consent was obtained from all patients.

### Patients with limbal stem cell deficiency

The study design was a prospective non-comparative case series comprising a sample size of 16 eyes from 16 patients with LSCD who had failed prior therapy. Patients were recruited between 2007 and 2011 and were referrals to the Corneal Unit at the Sydney Eye Hospital (Sydney, Australia), Eye Clinic at the Prince of Wales Hospital (Randwick, Sydney, Australia), or private rooms (Bondi Junction, Sydney). Patients with severe total or partial LSCD were included in the study, and LSCD was diagnosed clinically [10,23]. Clinical features that distinguished these patients included recurrent or persistent epithelial defects, corneal fibrovascular pannus, and chronic inflammation [10,23]. For partial LSCD, patients had failed all prior medical therapy and had at least 6 clock hours (180 degrees) of whorl-like epitheliopathy, opaque epithelium arising from the limbus, and superficial neovascularisation or conjunctivalisation or both [24,25]. Photographs were taken to document these features. Impression cytology was not performed as it can induce painful persistent epithelial defects with the risk of infection or increased inflammation or both [10]. Patients with total or partial LSCD were included if they had failed all prior therapy, which included autologous serum drops, preservative-free lubricants, therapeutic CL wear, limbal tissue allografts, HAM transplants, superficial keratectomy, and conventional corneal grafts. Table 1 summarises the baseline demographic features of our patients.

**Table 1:** **Patient demographics and pre-operative characteristics**

**Case. age at last follow-up,years/Sex**	**Eye, R/L**	**LSCD, unilateral/bilateral**	**Days in culture**	**LSCD, partial/total**	**Aetiology of LSCD**	**Previous procedures**	**Post-operative topical therapy**	**Post-operative immunosuppressive or systemic ocular therapy or both**
1. 40/M	R	B	14	T	Aniridia	Limbal tissue allograft + SK*, CE/IOL	FML, cellufresh tears, timolol	Minocycline 50 mg, mycophenolate mofetil 500 mg BD
2. 77/F	R	U	10	T	Primary acquired melanosis; multiple surgeries/topical mitomycin C therapy	Multiple conjunctival excisions	Predsol minims	Nil
3. 73/F	R	U	10	T	Recurrent melanoma; multiple surgeries	Multiple conjunctival excisions, cryotherapy, lid repair, HAM transplant	Cellufresh tears, dexa minims	Nil
4. 68/M	L	B	11	P	Query trachoma-induced	Ptosis repair	Cellufresh tears, dexa minims	Nil
5. 61/M	R	U	9	T	Chemical burn	Limbal tissue allograft*	Dexa minims	Doxcycline 100 mg
6. 65/F	R	B	16	T	Aniridia	2xPK*, cyclodiode laser	Dexa minims, timolol	Nil
7. 33/M	L	U	14	T	Chemical burn	HAM transplant + nexagon application*	Dexa minims	Nil
8. 50/M	L	U	14	T	Chemical burn	Ptosis repair, PK*, limbal tissue allograft + PK*, CE/IOL	Dexa minims, atropine sulphate 1% minims	Prednisone 80 mg in a tapering regime
9. 52/M	R	U	14	P	Query trachoma-induced	Removal of pseudopterygium	Dexa minims	Nil
10. 72/F	L	B	14	T	Aniridia	CE/IOL, ectropion repair, EDTA	Bimatoprost 0.03%, minims pilocarpine nitrate 2%, refresh tears	Acetazolamide 125 mg BD
11. 62/M	R	B	15	T	Multiple surgeries	PTK, PK (for corneal ectasia), CE/IOL	Dexa minims	Nil
12. 81/M	L	U	14	T	Chemical burn	Nil	Atropine sulphate 1% minims, timolol/latanoprost, dexa minims	Nil
13. 28/M	R	U	9	T	Chemical burn	BV diathermy	Dexa minims	Nil
14. 65/F	L	B	16	T	CL over-wear	PK*, CE/IOL, PK + limbal tissue allograft* + tarsorrhaphy, DSEK	Predsol minims, latanoprost/timolol, atropine sulphate 1% minims	Nil
15. 85/M	R	U	14	P	Limbal tumor excision; limbal surgeries	CE/IOL	Dexa minims, FML	Nil
16. 80/F	R	B	9	T	Cicatrising conjunctivitis; ocular surface toxicity from glaucoma medication	CE/IOL, blepharoplasty	Dexa minims, cellufresh tears, timolol/latanoprost	Nil

All patients were additionally prescribed minims chloramphenicol 0.5% post-operatively (not included in table). Asterisk (*) indicates previous surgical procedures to treat limbal stem cell deficiency (LSCD). Days in culture refers to the time to establish a reasonable number of cells on the contact lens before transplantation. BD, twice daily dosing; BV, blood vessel; CE/IOL, cataracts extraction/intraocular lens insertion; cellufresh tears, preservative free carboxymethylcellulose sodium 5 mg/mL; CL, contact lens; dexa minims, minims dexamethasone sodium phosphate 0.1%; DSEK, Descemet’s stripping endothelial keratoplasty; EDTA, ethylenediaminetetraacetic acid chelation therapy for band keratopathy; FML, flurometholone 1%; HAM, human amniotic membrane; PK, penetrating keratoplasty; predsol minims, minims prednisolone sodium phosphate 0.5%; PTK, phototherapuetic keratectomy;refresh tears, preservative free polyvinyl alcohol 1.4%; SK, superficial keratectomy.

### Cell culture, contact lens insertion, and post-operative follow-up

Multiple (two or three) autologous epithelial biopsies (approximately equal to 1 to 2 mm^2^) were taken from either the superior limbal region or superior conjunctival fornix of the contralateral eye under local anesthesia (Minims Tetracaine Hydrochloride 1%; Chauvin Pharmaceuticals, Bausch & Lomb, Kingston-Upon-Thames, UK) based on data suggesting that cells from the superior forniceal explants grow more effectively [26,27]. Serum was isolated from 20 mL of whole blood taken at the time of biopsy by standard venipuncture. Each biopsy was placed on the concave surface of a siloxane-hydrogel extended-wear CL (Lotrafilcon A; CIBA Vision, Duluth, GA, USA) in 24-well culture plates (Corning Inc., Corning, NY, USA) in Eagle’s minimum essential medium containing 10% autologous serum with antibiotic supplements as previously detailed [16,21]. Cultures were kept in an isolated incubator set to 37°C with 5% CO_2_, and growth was monitored daily with media changed on alternate days. When cells reached confluence (9 to 16 days), patients were scheduled for the procedure and the cell-coated CL transported to the operating theatre in growth media in cold storage (4°C to 10°C). This ensured that cell activity could be preserved in the event of delays in theatres. Cells emerging from tissues explanted on CLs have previously been phenotyped and shown to express several key ocular surface SC markers [21].

Patients with unilateral conditions had limbal and conjunctival biopsies harvested from separate sites, and patients with bilateral disease received cells from conjunctival biopsies. In patients with unilateral conditions, limbal biopsies were cultured on CLs; however, two patients (cases 5 and 15) had limbal biopsies that did not grow. Thus, these patients received ocular progenitor cells from conjunctival biopsies to avoid the risk of SC failure in the donor eye being induced by a repeat limbal biopsy.

Prior to insertion of the CL, 5% betadine was applied to the eye and a total superficial keratectomy, including removal of limbal epithelium, was performed to remove any irregular epithelium or pannus or both [21]. The CL with biopsy and expanding cells was inserted onto the patient’s ocular surface under topical anesthesia (Minims Benoxinate Hydrochloride 0.4%; Chauvin Pharmaceuticals, Bausch & Lomb). Penetrating keratoplasty (PK) was performed prior to CL insertion as indicated in patients who had endothelial failure with stromal edema (cases 6, 8, and 14) and stromal scarring (case 12) reducing vision. Post-operatively, patients continued to take prior systemic and topical therapy. For prophylaxis against infection, each patient was prescribed Minims Chloramphenicol 0.5% (Chauvin Pharmaceuticals, Bausch & Lomb), which was applied for 4 weeks. Twelve patients also received Minims Dexamethasone sodium phosphate 0.1% (Chauvin Pharmaceuticals, Bausch & Lomb) tapered over the course of 1 month. Two patients were continued on Minims Prednisolone sodium phosphate 0.5% (Chauvin Pharmaceuticals, Bausch & Lomb) (Table 1). The topical steroid regime was determined by the treating physician according to the degree of post-operative inflammation.

### Follow-up and assessment of outcome

Ophthalmological evaluations after the procedure were performed at days 1 and 7 and then at 1, 3, 6, and 9 months. Six-month follow-ups were scheduled during the following year and yearly visits thereafter. Each visit involved taking a medical history, recording ocular symptoms, imaging the eye, and performing Snellen’s test for best-corrected visual acuity (BCVA), slit-lamp examination, tear film assessment, ocular surface staining with fluorescein, and tonometry. Patients’ medical records were reviewed, and data were recorded on a proforma and entered into an electronic database. Success was defined as ocular surface stability and visual improvement unless otherwise limited by pre-existing or concomitant disease. Two authors (SW and SB) determined ocular surface stability from clinical examination, clinical photographs, and medical notes. Independent grading of each patient was also performed from clinical photographs by author ND. In alignment with previously published reports [4,14,23,28], restoration of corneal epithelium, reduction of neovascularisation, and the absence of recurrent or persistent epithelial defects (PEDs) were the parameters used to determine ocular surface stability. Corneal epithelialisation was defined on the basis of transparency without epithelial defects on slit-lamp examination and the absence of abnormally high fluorescein permeability. Partial success was defined as improvements in subjective ocular symptoms or BCVA or both with a stable central corneal epithelium and no PEDs despite the presence of peripheral epithelial whorl-staining or recurrent vascularisation, even if not as extensive as at the time of admission [14]. Treatment failure was defined as recurrence of LESC failure with conjunctivalisation of the ocular surface.

### Statistical analysis

Survival probability of grafts was analyzed by Kaplan-Meier and the log-rank test. Graft survival began at the time of transplant, and an event was defined as failure or success at the last follow-up. Descriptive statistics were used to summarise all continuous and categorical variables. Analyses were performed by SPSS 21.0 software (IBM SPSS Statistics, version 21; IBM Corporation, Armonk, NY, USA).

## Results

### Cell culture and growth of biopsies

**Figure 1:**
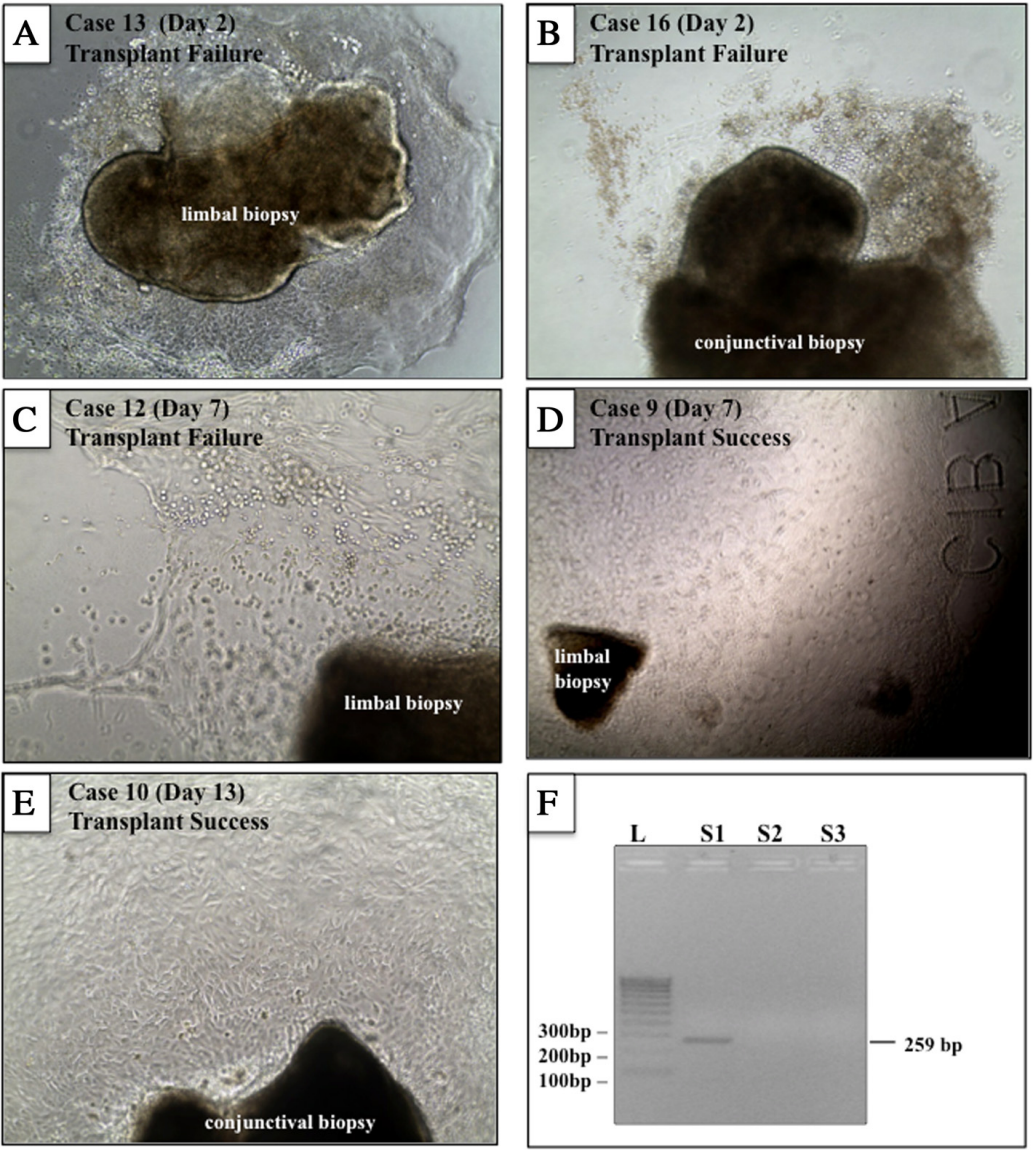
**Cell growth from limbal and conjunctival biopsies.** Phase-contrast images of limbal **(A, C, D)** and conjunctival **(B, E)** biopsies excised from patients with limbal stem cell deficiency and cultured over a specific period (see panel label for case identification number and time in culture). Although cultures displayed ample proliferation activity, some grafts failed **(A-C)** whereas others were successful **(D and E)** at last follow-up. A representative polymerase chain reaction for mycoplasma **(F)** on conditioned media derived from cultured cells from patient 12 (S2) is shown. S1 (positive control) shows a band at 259 base pairs (bp), and S3 is a negative control.

Cells began to emerge as early as 2 days in culture irrespectively of whether limbal or conjunctival tissue biopsies were used (Figure 1A and B). Eventually, a halo of cells of similar morphology surrounded each limbal or conjunctival biopsy, and the migratory front of expanding cells reached the edge of the CL by 9 to 16 days post-explanting (Figure 1C-E). In four (25%) out of 16 patients, a second set of biopsies was harvested for culture purposes as insufficient growth developed from the first. No signs of SC failure were observed in any donor eye. No other complications were noted in regard to the culture component. Prior to transplantation, an aliquot of media from each culture was tested for mycoplasma contamination; however, none returned a positive reading (Figure 1F).

### Patient characteristics

Sixteen eyes from 16 patients were enrolled in the study between 2007 and 2011. The mean age at follow-up was 62 ± 17 years (range of 28 to 85), and the male-to-female ratio was 10:6. Table 2 summarises the post-operative characteristics and outcomes of patients enrolled in the trial. The most common aetiologies were chemical burns (31%), iatrogenic causes (25%), and aniridia (19%). Other underlying aetiologies were trachoma-induced LSCD (cases 4 and 9), CL over-wear (case 14), and cicatrising conjunctivitis due to ocular surface toxicity from glaucoma medications (case 16).

**Table 2:** **Post-operative characteristics and outcomes of ocular surface stem cell transplantation via contact lens delivery**

**Case**	**Type of graft**	**Follow-up, years**	**Complications during cell culture**	**Procedure performed in trial**	**Subsequent procedures**	**Pre-operative visual acuity**	**Post-operative visual acuity**	**Ocular surface**	**Notes**	**Result**
1	C	5.75	CL rolled under superior lid, required insertion of second CL	SCT + SK	-	6/60 + 1	6/45	Central clear, peripheral pannus	Glaucoma, DE, subepithelial scarring	S
2	L	5.67	biopsy retained in cornea	SCT + SK	DSEK (endothelial decompensation), CE/IOL	6/18-1	6/60	Central clear, peripheral vascularisation	Endothelial decompensation	S
3	L	5.33	Nil	SCT + SK	-	CF at 0.2 m	LP	Central clear, peripheral vascularisation	Stromal scarring, corneal stromal opacity, cataracts	S
4	C	2.92 (lost to follow-up)	Nil	SCT + SK	-	6/45	6/36	Central clear, superior peripheral pannus	Subepithelial scarring	S
5	C	4.67	Second biopsy, no/poor growth	SCT + SK	-	CF at 0.5 m	1/60	Central clear, peripheral pannus	Stromal scarring	S
6	C	4.17	Second biopsy, no/poor growth	SCT + PK	PK + HAM graft (failed transplant), blepharoplasty, CE/IOL, Molteno implant	HM	6/60	PED	Corneal astigmatism, glaucoma, DE	F
7	L	3.25	Second biopsy, no/poor growth	SCT + SK	-	CF at 1 m	CF at 1 m	PED, recurrent corneal vascularisation	Subepithelial scarring	F
8	L	2.58	Nil	SCT + PK	YAG	HM	0.5/60	PED	Graft astigmatism, stromal scarring	F
9	L	0.75	Small defect created on removal of CL	SCT + SK	-	6/6	6/6	Central clear	Corneal astigmatism, subepithelial scarring	S
10	C	2.42	Nil	SCT + SK	-	CF at 2 m	6/90	Central clear, ulcers from toxicity of glaucoma meds	DE, severe glaucoma, progressive corneal oedema, stromal scarring	S
11	C	2.17	Nil	SCT + SK + EDTA + BV diathermy	-	2/60.	1.5/60	Central clear, pannus to graft-host junction	DE, postoperative endophthalmitis (prior PTK), corneal ectasia, subep scarring	S
12	L	2.25	Second biopsy, no/poor growth	SCT + PK + CE/IOL	-	3/60	CF	Irregular epithelium, graft opacity, PEEs, PEDx2 on graft	DE, glaucoma, ocular surface toxicity, stromal scarring	F
13	L	2	Nil	SCT	HAM transplant (corneal ulcer) + tarsorrhaphy, PK (corneal perforation)	HM	HM	Swirled epithelium, PED, recurrent corneal vascularisation	Stromal scarring	F
14	C	1.92	Nil	SCT + PK		HM	6/90	Central clear, mild subepithelial haze, <2 clock hours of superior-temporal whorl-like corneal staining	DE, graft astigmatism, glaucoma, stromal scarring	PS
15	C	1.67	Nil	SCT + SK	EDTA	6/36	6/90	Central clear	LK, stromal scarring	S
16	C	1.67	Nil	SCT + SK	Cyclodiode laser	2/60.	1/60	Corneal ulcer, PED	DE, stromal scarring, glaucoma	F

BV, blood vessel; C, conjunctival; CE/IOL, cataract extraction/intraocular lens; CF, counting fingers; CL, contact lens; DE, dry eye; DSEK, Descemet’s stripping endothelial keratoplasty; EDTA, ethylenediaminetetraacetic acid chelation therapy for band keratopathy; F, failure; HAM, human amniotic membrane; HM, hand movements; L, limbal; LK, lipid keratopathy; LP, light perception; PED, persistent epithelial defect; PEEs, punctate epithelial erosions; PK, penetrating keratoplasty; PS, partial success; PTK, phototherapuetic keratectomy; S, success; SCT, stem cell transplant; SK, superficial keratectomy; YAG, yttrium aluminium garnet capsulotomy.

### Ocular surface stability

**Figure 2:**
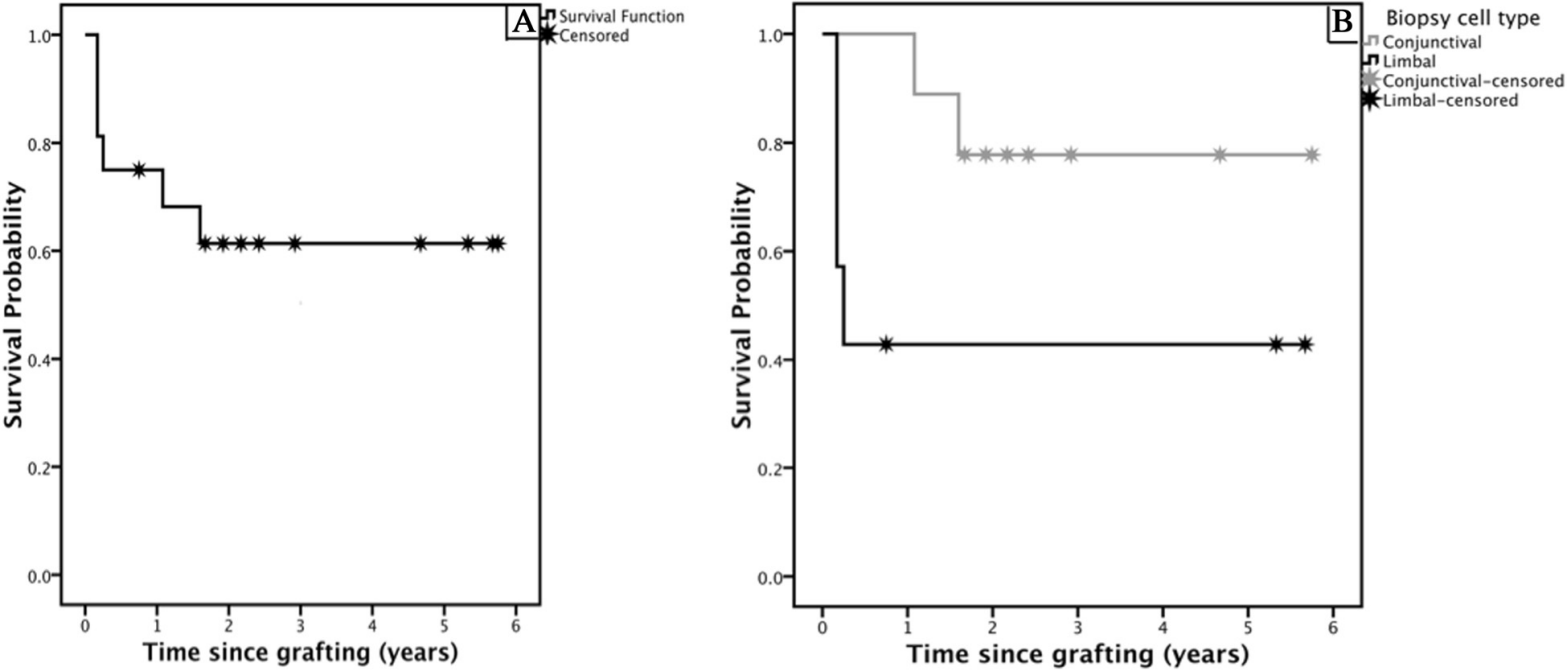
**Kaplan-Meier survival curve.** Sixteen eyes of 16 patients who underwent ocular surface epithelial transplantation via contact lens delivery were assessed for graft survival. **(A)** Total or partial success was attained in 63% of the cases. **(B)** Stratified by epithelial origin of the cell graft total or partial success was attained in 78% of cases after transfer of conjunctival biopsies (n = 9) and 43% of cases after transfer of limbal biopsies (n = 7).

**Figure 3:**
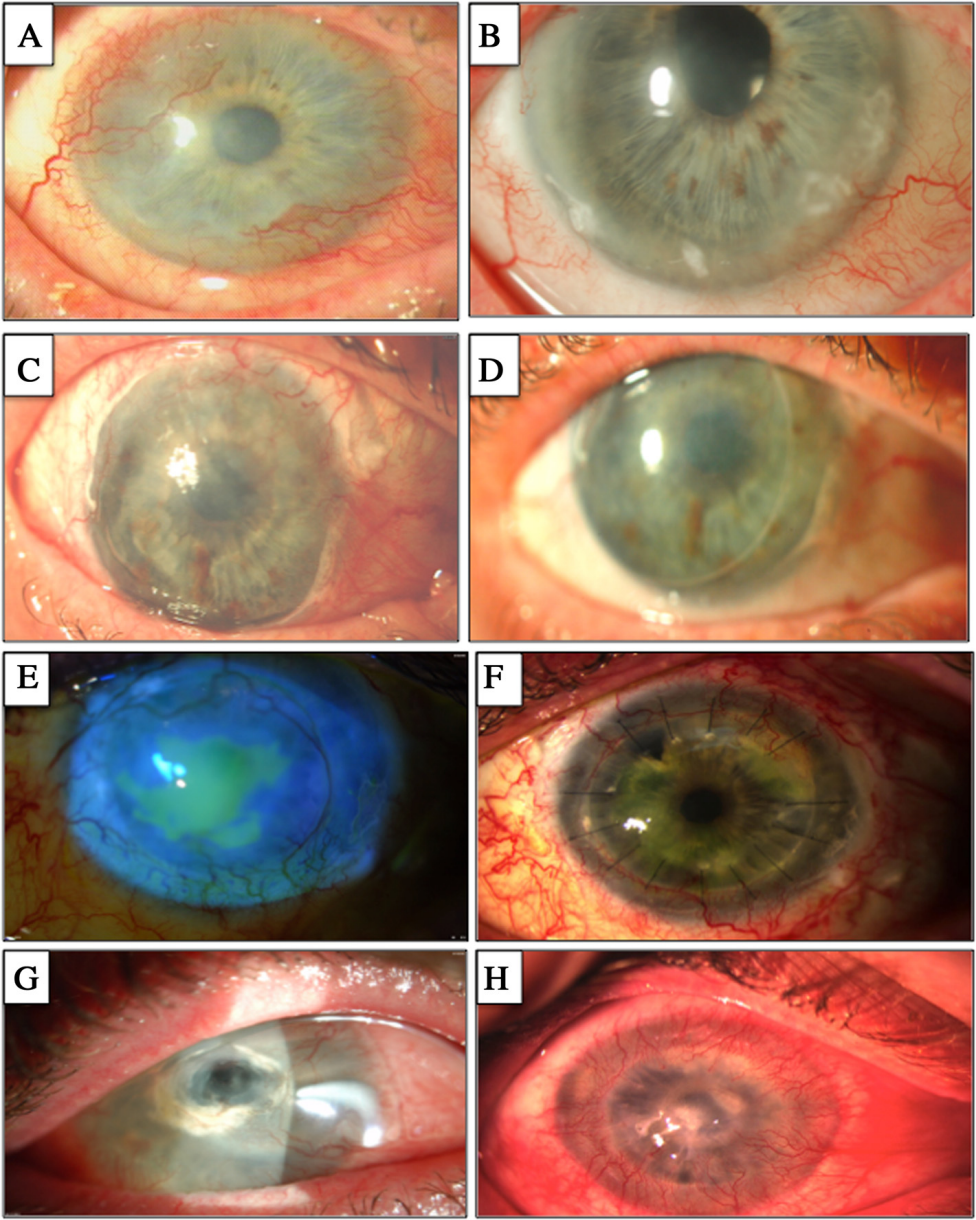
**Clinical features of patients who underwent cell transplantation.** Slit-lamp photographs of successful transplants in patient 2 **(A, B)** and patient 14 **(C, D)** before treatment **(A, C)** and at last follow-up—**(B)** 5.6 years and **(D)** 1.9 years—with restoration of an intact transparent corneal epithelium and reduced vascularisation **(B)**. Notably, a localised region of superior-temporal whorl-like staining (obscured by the eyelid) was evident on clinical examination in patient 14 **(D)**. Slit-lamp photographs of failed transplants in patient 8 **(E, F)** and patient 13 **(G, H)** before treatment **(E, G)** and at last follow-up—**(F)** 2.6 years and **(H)** 2 years—with an irregular corneal epithelium and recurrence of epithelial defects.

Restoration of ocular surface stability was seen in 12 eyes (75%) at 1 year and 11 eyes (69%) at 2 years with a cumulative survival of 63% after a median follow-up time of 2.5 ± 1.2 years (range of 0.8 to 5.8) (Figures 2A and 3A and B). Biopsies of conjunctival origin had a higher cumulative survival (78%) compared with the survival rate displayed by limbal biopsies (43%); however, this was not a statistically significant difference as assessed by the log-rank test (Figure 2B, *P* = 0.06). The outcomes of three of these patients (cases 1 to 3) at the 1-year follow-up were published previously [20], and all continue to maintain a stable ocular surface at the 5- to 6-year follow-up. Case 1, though still with visual improvement from baseline, has recently developed central subepithelial scarring and increasing epithelial irregularity. A small area of localised inferonasal band keratopathy adjacent to the limbus developed in case 2, with endothelial decompensation, and has occasionally developed local ulceration associated with the band keratopathy. One eye (case 14) was deemed a partial success, having achieved a transparent central corneal epithelium with no recurrent ulcers or PEDs or both, complete resolution of ocular pain, and improved visual acuity (hand movements to 6/90). This was despite a localised region (fewer than 2 clock-hours) of superior-temporal whorl-like corneal staining, which remained stable at the 2-year follow-up period (Figure 3C and D). When analysed according to the aetiology of LSCD, patients with iatrogenic causes experienced a 100% success rate (four out of four cases) compared with a 20% success (one out of five cases) for patients with chemical burns. Aniridia was associated with a 67% success rate (two out of three cases), and patients with trachoma were both deemed successful. When analysed according to the severity of LSCD, the three patients with partial LSCD experienced a 100% success rate and the patients with total LSCD experienced a 54% success rate.

### Visual acuity and ocular symptoms

In 90% of patients deemed successes or partial successes, ocular symptoms (pain, burning, and photophobia) resolved completely. Pre-existing corneal scarring or concomitant ocular disease limited visual improvement in most patients (Table 2). Fifty percent of patients who achieved a successful outcome attained improved visual acuity (at least one line) and the biopsies in each of these five patients were all of conjunctival origin. Twenty percent maintained their pre-operative visual acuity (for example, patient 9 maintained a BCVA of 6 out of 6 after receiving cells from a limbal biopsy, and patient 11 maintained a BCVA of 2 out of 60 after a conjunctival biopsy). Thirty percent of the patients deemed successes experienced a decline in BCVA by 4, 3, and 1 line (cases 2, 15, and 3, respectively). The BCVA of patient 2 declined 3 years after SC transplantation because of endothelial decompensation and was subsequently treated with Descemet’s stripping endothelial keratoplasty (DSEK). The decline in visual acuity of patient 3 was attributed to progressive cataract (untreated because of patient preference and corneal scarring limiting visual potential), and patient 15 to lipid keratopathy. Though deemed failures due to recurrence of epithelial defects, patients 6 and 8 both achieved improvements in their BCVA attributed to successful treatment of concomitant ocular disease (Table 2). By excluding these patients with confounding co-morbidities from analysis, we demonstrated that 71% of the remaining seven patients with a conjunctival biopsy had an improved BCVA and that 33% had a stable BCVA. This compares favourably with 75% of the remaining four patients with limbal biopsies having a stable BCVA and 25% a reduced BCVA.

### Characteristics of failed transplants

Failed transplants were characterised by recurrence of conjunctivalisation, vascularisation, corneal epithelial defects or a combination of these (Figure 3F and H). Of the failures, the underlying aetiology was chemical burns in 67% (four out of six cases), and aniridia and cicatrising conjunctivitis were the cause in the other two patients. Of the failures, 50% had a second set of biopsies harvested prior to the cell transfer therapy because of insufficient growth from the first explants. In 67% of failures, biopsies were of limbal rather than conjunctival origin. Most failures (67%) occurred within the first 4 months after transplantation; two eyes (cases 6 and 16) failed in the following 1 to 2 years, both complicated by ocular surface toxicity due to glaucoma medications. Fifty percent of the failures had prior grafts (to treat LSCD) that had also failed.

## Discussion

This study found that transplantation of autologous limbal and conjunctival epithelial cells via CL delivery successfully restored the ocular surface in 63% (10 out of 16 cases) of patients with LSCD at a median follow-up time of 2.5 ± 1.2 years (range of 0.8 to 5.8). The reported success rate of cultivated autologous limbal epithelial cell transplantation (LSCT) ranges from 33% to 100% with a mean of 75% at 2-year follow-up [4,6,10,23,28-31]. Although our results were lower than the overall mean success rate [4,6], they are within the range reported by others [4,6,10,23,28-31]. Furthermore, recent clinical trials [10,32] have shown that repeat autologous cultivated LSCT following failed primary transplantation surgery successfully replenishes the ocular surface. As our technique is repeatable, a second cell transfer via CL in failed transplants could also increase the success rate of our technique in future studies. The variability in results from trial to trial could be attributed to patient selection and pre-operative condition; the majority of patients in our study failed all prior treatment regimens, including corneal grafts and limbal tissue transplants, and some even had coexistent ocular disease.

Although autologous limbal epithelial biopsies are the tissue of choice when expanding SCs for transplantation, the limited availability of limbal tissue particularly in cases of bilateral disease has necessitated the use of alternative tissue sources. In our study, autologous conjunctiva was used as a source of epithelial SCs for transplantation in cases of bilateral disease, and successful outcomes were reported in seven (78%) out of nine patients, unexpectedly higher than the 43% success rate for cells of limbal origin (Figure 2B). Although these results did not reach significance, the higher success rates in patients receiving conjunctival compared with limbal cells could be attributable to the slightly larger biopsies that were obtained from conjunctival tissue. Larger biopsies were harvested from the conjunctiva as there was no risk of inducing SC failure in the donor eye. Notably, this explanation is purely speculative as conjunctival cells in culture did not grow faster than the limbal equivalents and there are no published reports indicating that cells from larger biopsies are associated with improved patient outcomes. Additionally, it has been demonstrated that detachment of limbal explants from their scaffolds results in unsuccessful primary cultures [33], potentially explaining the failure of cell growth from limbal explants in cases 5 and 12.

In 25% of our patients, cells failed to grow from explants and a second set of biopsies was harvested before successful *ex vivo* expansion. Although many studies do not disclose the number of detachments, Sangwan and colleagues [31] (2011) reported successful cellular growth from tissue explants in all 200 eyes when cultured on HAM, suggesting that a synthetic CL scaffold may not be as effective as a native substrate for expansion. The ability to make direct comparisons with their study, however, is hindered by the heterogeneity of disease. Whereas patients in the study by Sangwan and colleagues were diagnosed with unilateral ocular surface burns, 44% of the patients in the present study had bilateral LSCD and many cases also had had previous surgeries to the donor eye, potentially impacting the proliferative capacity of harvested cells. Additionally, the culture technique of Sangwan and colleagues, which involved shredding presumably larger segments of donor limbal tissue into small pieces and explanting these over the substrate, could have contributed to the higher success rate of their culture system. Regardless, our study addresses the challenge of biopsy detachment since multiple autologous samples were obtained and the CL substrate is easily available, allowing more than one culture to be initiated with minimal difficulties.

In relation to lineage origin, the conjunctival epithelium is perhaps the cell type most closely related to the corneal epithelium [34]. Kawasaki and colleagues [35] found a population of keratin-12-positive cells in the conjunctival epithelium, presumed to be ectopically residing corneal epithelial cells. Additionally, Majo and colleagues [36] discovered that porcine corneal and conjunctival holoclones shared an ocular gene expression profile, supporting our proposition that conjunctival and limbal epithelial cells can be interchanged. Notably, their results have raised controversy on multiple levels; however, they noted two differentially expressed genes between these epithelia from over 20,000 assessed. Although the precise mechanisms of how conjunctival cells re-establish the ocular surface are not known, the conjunctiva contain its own SCs that may have the ability to transdifferentiate when exposed to corneal-specific signals [37,38]. For example, Shapiro and colleagues [39] found that, after 4 to 5 weeks, transplanted conjunctival epithelial cells morphologically resembled corneal epithelium. It should be noted, however, that these findings are controversial, and subsequent studies showed that transplanted conjunctival cells retained lineage-specific features [40,41]. Despite this, transplanted conjunctival epithelium has been shown to successfully regenerate the ocular surface in rabbit models of total LSCD [38,42,43] as well as in patients with LSCD [44-46]. Notably, end-stage limbal stem cell failure is characterised by conjunctivalisation of the ocular surface. However, the conjunctival cells transferred in our study were epithelial in origin, and isolated from an area of conjunctiva that was not affected by disease, which may have accounted for their transparency after engraftment. Further conjunctival epithelial cells alone, without the vascularised conjunctival stroma were utilised for our patients. The present study demonstrates the effectiveness of conjunctival-derived progenitor epithelial cells in transplants, suggesting that even if conjunctival cells do not transdifferentiate, they may acquire a corneal-like phenotype under the culture conditions provided.

Although the primary aim of restoring corneal epithelial integrity and thus resolving ocular discomfort was achieved, improved visual acuity as the secondary outcome measure was attained in 50% of the successful transplants with at least a one-line improvement in BCVA and 20% maintaining their pre-operative visual acuity (Table 2). This was attributed to the majority of patients having pre-existing corneal scarring or concomitant ocular disease limiting vision or both. Most (90%) patients with successful transplants reported complete resolution of their ocular symptoms (burning, photophobia, or discomfort), and one patient (case 11) complained of some (albeit reduced) discomfort, potentially attributable to his coexisting dry eye.

The cause of graft failure in patients with LSCD is poorly understood. Li and colleagues [47] have recently shown that limbal niche and stromal cells are important in supporting LESCs; thus, damage to the stromal niche microenvironment could contribute to failures in some patients since SC transfer does not address this anatomical and structural modification. Curiously, we and others [48] observed a higher failure rate in patients with chemical injuries, the cause of which is unknown but could be due to excessive niche damage. Furthermore, of the six failed transplants in our study, four failed within the first 4 months and two eyes failed in the following 1- to 2-year period, both complicated by ocular surface toxicity due to glaucoma medications. Additional investigations are required to determine whether glaucoma medication could be revised prior to and during a specific period post-cell therapy to increase the survival probability of grafts in patients with coexisting severe glaucoma. Indeed, since our study commenced, a wider range of glaucoma medications with less potential for ocular surface toxicity have become available [49].

Minor complications occurred with CL insertion and removal in two patients. In patient 1, we noted that the CL rolled under the superior lid. However, since our procedure involves harvesting multiple biopsies, a second cell-laden CL was readily available and this was inserted over the patient’s cornea the following day. The option of a replacement graft is attractive and advantageous as it reduces the need to re-biopsy, re-culture cells, re-schedule the procedure, and delay treatment for the patient. In case 9, a small defect occurred upon removal of the CL with stripping of superficial corneal epithelial cells; however, this resolved within 24 hours and was not associated with any long-term sequelae. In a third patient (case 2), the biopsy integrated onto the patient’s cornea and the transplant was deemed a success at 5.7 years. Retention of the biopsy on the ocular surface was a positive prognostic factor in this patient, but if integration occurred over the visual axis, sight could have been affected. Although it has been recognised that CL wear can be associated with severe limbal SC failure [24], this occurs with long-term wear; the total time of lens wear in our patients was 2 weeks. Notably, irrespectively of the type of graft, once the lens was removed from the patients’ ocular surface, it contained few remnant cell colonies [21].

We acknowledge several limitations of our study. Firstly, our investigation included a small sample size and a heterogeneous patient population. The differing aetiologies, pre-operative condition, and concomitant ocular morbidities limit the potential to make direct comparisons with previously published studies [48,50]; however, most reports share similar constraints. Furthermore, with the exception of the case report by Ang and colleagues [51], who compared the efficacy of conventional and cultivated LSCT, almost no direct comparative studies for LSCT in human subjects have been published. It is worth noting that success rates differ significantly between patient groups with different aetiologies of LSCD and that further studies investigating specific patient subtypes could reveal, for example, particular effectiveness of CL delivery for patients with iatrogenic-induced LSCD. Furthermore, we included both total and partial LSCD as patients in both groups had failed prior therapy. Ideally, a control group should have been included; however, owing to the severity and progressive nature of LSCD, it is unethical not to treat these patients. The lack of a standardised framework for the diagnosis and grading of LSCD is also a limitation of our study and is a major concern of most published work in this field [50,52]. An alternative objective scoring system could be developed on the basis of impression cytology. However, this procedure has not been routinely performed in clinical trials of LSCD [50] as it does not significantly change the clinical diagnosis and exposes patients to unnecessary pain and the risk of developing epithelial defects [10]. Symptom-based questionnaires are also flawed in the subjectivity of self-reporting and have not been validated for patients with LSCD. Another limitation of the present study is the inability to directly trace the fate of transplanted cells. The mechanism by which SC transplantation regenerates the corneal epithelium is not well understood, and there is controversy surrounding whether transplanted cells actually replenish the SC reserve or revive any remaining quiescent SCs [10,29]. To address these critical questions, one could perform genetic lineage tracing [53] but these studies would need to be performed in laboratory animals, whereby marked SCs from transgenic mice could be transplanted into wild-type recipients and their long-term fate and function determined. We have made progress in this area and recently established a unique transgenic model whereby progenitor cells and their progeny are traceable in live mice in real time [54].

The strengths of our study include its prospective nature and length of follow-up. Our current findings substantiate our earlier report [21] and confirm that SCs transplanted via our novel CL delivery technique are maintained for longer than 1 year, and successful outcomes were recorded at a maximum follow-up of 5.8 years. The greatest advantage of our approach is its autologous xeno-free nature and the benefits in cost-effectiveness and accessibility over other transplantation strategies. Additionally, CLs have been shown to adsorb growth factors from serum and may act as a slow-release device for SC-promoting factors at least during the implantation period [55,56]. Recently, surface modifications to CL polymers were demonstrated to enhance the loading and transfer capacity of corneal epithelial cells to wounded rabbit corneas [19,20].

## Conclusions

In this study, we have shown that our technique of ocular surface epithelial SC transplantation is a viable and promising alternative to current approaches, successfully regenerating a healthy ocular surface in patients with LSCD at short- to mid-term follow-up in 63% of our patients. This is consistent with similar studies using alternative transplantation methodologies but our technique does not expose the grafts to foreign human biological or xenogeneic materials.

## Abbreviations

BCVA: best corrected visual acuity
CL: contact lens
HAM: human amniotic membrane
HREC: Human Research Ethics Committee
LESC: limbal epithelial stem cell
LSCD: limbal stem cell deficiency
LSCT: limbal stem cell transplantation
PED: persistent epithelial defect
SC: stem cell
SESLHD: South Eastern Sydney Local Health District Human Research Ethics Committee
